# Who is in the near market for bicycle sharing? Identifying current, potential, and unlikely users of a public bicycle share program in Vancouver, Canada

**DOI:** 10.1186/s12889-018-6246-3

**Published:** 2018-11-29

**Authors:** Kate Hosford, Scott A. Lear, Daniel Fuller, Kay Teschke, Suzanne Therrien, Meghan Winters

**Affiliations:** 1 Faculty of Health Sciences, Simon Fraser University, 8888 University Drive, Burnaby, BC V5A1S6 Canada; 2 Centre for Hip Health and Mobility, 2635 Laurel Street, Vancouver, BC V5Z 1M9 Canada; 3 Division of Cardiology, Providence Health Care, 1081 Burrard Street, Vancouver, BC V6Z 1Y6 Canada; 4 School of Human Kinetics and Recreation, Memorial University of Newfoundland, Physical Education Building, St. John’s, Newfoundland A1C 5S7 Canada; 5 Department of Community Health and Humanities, Faculty of Medicine, Memorial University of Newfoundland, St. John’s, Newfoundland A1B 3V6 Canada; 6 School of Population and Public Health, University of British Columbia, 2206 East Mall, Vancouver, BC V6T 1Z3 Canada

**Keywords:** Bicycling, Bicycle share, Active transportation, Social marketing

## Abstract

**Background:**

Public bicycle share programs in many cities are used by a small segment of the population. To better understand the market for public bicycle share, this study examined the socio-demographic and transportation characteristics of current, potential, and unlikely users of a public bicycle share program and identified specific motivators and deterrents to public bicycle share use.

**Methods:**

We used cross-sectional data from a 2017 Vancouver public bicycle share (Mobi by Shaw Go) member survey (*n* = 1272) and a 2017 population-based survey of Vancouver residents (*n* = 792). We categorized non-users from the population survey as either potential or unlikely users based on their stated interest in using public bicycle share within the next year. We used descriptive statistics to compare the demographic and transportation characteristics of current users to non-users, and multiple logistic regression to compare the profiles of potential and unlikely users.

**Results:**

Public bicycle share users in Vancouver tended to be male, employed, and have higher educations and incomes as compared to non-users, and were more likely to use active modes of transportation. The vast majority of non-users (74%) thought the public bicycle share program was a good idea for Vancouver. Of the non-users, 23% were identified as potential users. Potential users tended to be younger, have lower incomes, and were more likely to use public transit for their main mode of transportation, as compared to current and unlikely users. The most common motivators among potential users related to health benefits, not owning a bicycle, and stations near their home or destination. The deterrents among unlikely users were a preference for riding their own bicycle, perceived inconvenience compared to other modes, bad weather, and traffic. Cost was a deterrent to one-fifth of unlikely users, notable given they tended to have lower incomes than current users.

**Conclusion:**

Findings can help inform targeted marketing and outreach to increase public bicycle share uptake in the population.

**Electronic supplementary material:**

The online version of this article (10.1186/s12889-018-6246-3) contains supplementary material, which is available to authorized users.

## Background

Cities often implement public bicycle share programs as a way to help shift populations towards active and sustainable modes of transportation. By making bicycles available at docking stations throughout a city, these programs increase access to bicycles, especially for those who do not own a bicycle. However, public bicycle share programs are not used equally by all segments of the population. In many cities, program members tend to be male, Caucasian, employed, and have higher educations and incomes compared to the general population [1]. This has raised concerns that public bicycle share programs are further disadvantaging populations that may already experience inequitable access to transportation options [2, 3]. In addition, the majority of bicycle share trips replace trips previously made by walking or public transit, indicating that bicycle share appeals to people who already use active and sustainable modes of transportation [4–6]. In order to meaningfully contribute to creating a population level mode shift towards active and sustainable transportation, and to do so equitably, public bicycle share programs need to appeal to a broader population.

Social marketing is one approach to increase equitable access to public bicycle share and promote more widespread uptake. This approach involves the use of marketing concepts and strategies to influence behaviour change, and has commonly been used in public health to influence a number of behaviours including physical activity, drinking and driving, and smoking [7]. Social marketing has also been used for other transportation modes, such as bicycling, car sharing, and public transit [8–10].

A key aspect of social marketing is tailoring marketing and outreach strategies to segments of the population that share similar desires, attitudes, demographic characteristics or behaviours [7]. In the case of public bicycle share programs, this requires an understanding of who the users and non-users of these programs are, their attitudes towards such programs, and specific motivators and deterrents to program use.

A number of previous studies focus on understanding users of public bicycle share programs and motivators and deterrents to use [11–15]. Investigations of non-users of public bicycle share programs are less common, and often focus on understanding specific segments of the population (e.g., low income residents) or have small sample sizes that are not representative of the general population [1, 16–18]. Moreover, studies rarely stratify non-users based on their interest in using public bicycle share. Better understanding of the potential and unlikely users of public bicycle share programs along with their motivators and deterrents can provide evidence for bicycle share demand across socio-demographic groups and can serve as valuable data for social marketing efforts by public bicycle share operators and cities with the goal of increasing public bicycle share uptake at the population level.

To better understand the market for public bicycle share, this exploratory study examined the socio-demographic and transportation characteristics of current, potential, and unlikely users of the Vancouver public bicycle share program and identified specific motivators and deterrents to public bicycle share use. In the discussion, we provide examples of social marketing strategies that may help to increase uptake of public bicycle share, particularly amongst the potential user group.

## Methods

### Context

The City of Vancouver has a population of 630,000 people [19]. Bicycling for transportation is growing in popularity, with bicycling commute to work mode share estimated at 6.1% in 2016, up from 4.4% in 2011 [19, 20]. Compared to many North American cities, Vancouver has an extensive bicycle network with over 320 km of bicycle routes throughout the city. Vancouver’s public bicycle share program, Mobi by Shaw Go, launched in the summer of 2016 in the downtown core with 23 stations and 250 bicycles. As of fall 2017, the program has 122 stations and ~ 1200 bicycles with a service area of 17 km^2^, and has been used for more than 680,000 trips [21]. There are three Mobi by Shaw Go passes available for purchase: 24-h ($9.75), 3-month ($75), and annual ($129), which provide unlimited 30-min bicycle share trips.

### Data

We used data from two cross-sectional surveys. For current bicycle share users (required to be ≥18 years), we used an online Mobi member survey distributed to all annual and monthly members enrolled as of September 9, 2017 (survey dates: September 22–October 6, 2017, *n* = 1400, 29.4% response rate). To characterize the potential market for public bicycle share (potential users and unlikely users), we used a population-based survey of Vancouver residents (≥18 years) recruited through an online panel using age and sex quotas (October 13–31 2017, *n* = 966, 15.6% response rate). The survey was described as exploring transportation choices in Vancouver and did not mention the ‘Vancouver public bicycle share program’ to avoid biasing participation. Both surveys included questions on individual and household demographics, transportation access, transportation behaviour, public bicycle share use or likelihood of use, and motivators and deterrents to public bicycle share use (see Additional file 1 and Additional file 2 for a complete list of survey questions). The Simon Fraser University Research Ethics Board approved all study procedures and respondents provided written informed consent.

### Measures

We considered all respondents from the Mobi member survey that had used the program at least once to be “current users” of public bicycle share. And we considered all respondents from the Vancouver population survey who had not used the program to be “non-users” of bicycle share, and further categorized this group as either potential or unlikely users based on their response to the question, “How likely would you be to use public bike share in Vancouver at some point in the next year, given that station locations are accessible to you?” We categorized respondents who selected “very likely” or “somewhat likely” as potential bicycle share users, and respondents who selected “not likely” or “not all likely” as unlikely users.

We examined socio-demographic and travel characteristics that are potentially related to public bicycle share use, and were available in both the Mobi member and Vancouver population survey datasets. Variables included: individual demographics (sex, age, education, employment status, place of birth); household demographics (household income, having children at home); transportation access and behaviour (car access, car share membership, bicycle access, primary mode of transportation, bicycled in the past year, perceived safety of bicycling in Vancouver); location (living and/or working within 500 m of a Mobi by Shaw Go docking station); and perception of the Vancouver public bicycle share program.

We identified motivators and deterrents to using public bicycle share from two Vancouver population survey questions. We asked “potential users” of the bicycle share program to select all the reasons that would influence their decision to use the program from a 14-item list. Similarly, we asked “unlikely users” to select all the reasons that would influence their decision to not use the program from an 18-item list. The items listed were based on input from Mobi by Shaw Go and the City of Vancouver and from motivators and barriers to public bicycle share use identified in previous studies [14, 16, 22].

### Analysis

We applied weights to the Vancouver population survey respondent age and sex strata to match those of the 2016 Canadian census data for the city. We excluded “current users” from the Vancouver population survey due to the small number (*n* = 57) and different survey methodologies between the population survey and Mobi member survey.

In the first part of the analysis, we used descriptive statistics from the member and population surveys to contrast the socio-demographic and travel characteristics of current public bicycle share users with non-users of the program. We focused on percentage differences of at least 5% and trends across categories. In the second part of the analysis, we used logistic regression to identify variables that are associated with being a potential user of bicycle share, compared to an unlikely user, using data from the population survey. For the multivariable model, we used backward stepwise regression and selected the model with the lowest Akaike Information Criterion (AIC) value. The final multivariable model included age, employment status, place of birth, annual household income, car share membership, primary mode of transportation, bicycled in the past year, and perceived safety of bicycling. Finally, we present potential motivators to using the program among potential users and potential deterrents among unlikely users from the population survey, ranked by the percentage of respondents that selected the motivator or deterrent. All statistical analyses were conducted in R version 3.4.3.

## Results

In total, 1400 respondents completed the Mobi member survey and 966 respondents completed the Vancouver population survey. Of the 1400 Mobi member survey respondents, we excluded 34 that had not yet used the program and 94 with missing demographic data. Of the 966 population survey respondents, we excluded 53 who lived outside of the city of Vancouver (the study area), 35 with missing demographic data, 57 who used the public bicycle share program previously, and 29 who did not indicate a likelihood of using the program (i.e., responded “Don’t know” or “I prefer not to answer”). Our final analytic sample included 1272 current users and 792 non-users, of whom 182 were potential users (23%) and 610 were unlikely users (77%).

### Current users compared to non-users

Table 1 presents characteristics of current, potential, and unlikely users of the Vancouver public bicycle share program. Current users were disproportionately male (58.3%) and between the ages of 25–54 (85.6%), and more likely to be employed (90.5%) and have a graduate degree (34.8%). Current users were more likely to have household incomes >$150,000 compared to potential and unlikely users (27.2% compared to 10.0% and 11.2%, respectively), and potential users had lower incomes compared to the other two groups. Responses across transportation variables indicate that current users are the most oriented towards active modes of transportation. Current users were more likely to have a car share membership (67.7%) and a personal bicycle (69.8%), report walking or bicycling as their primary mode of transportation (45.2%) and perceive bicycling to be safe (79.3%). Most current users either lived or worked inside the bicycle share service area (92.0%), compared to 58.2% of potential users and 49.7% of unlikely users.

**Table 1:** Characteristics of current users and non-users of the Mobi by Shaw Go public bicycle share program in Vancouver, from a sub-sample of the 2017 Mobi member survey (*n* = 1272) and 2017 Vancouver population survey (*n* = 792)

	Current Users	Potential Users	Unlikely Users
	*n* = 1272	*n* = 182	*n* = 610
	n (%)	Weighted n (%)	Weighted n (%)
Demographics			
Sex, Female	530 (41.7)	92.5 (50.8)	318.0 (52.1)
Age			
18–24	42 (3.3)	22.4 (12.3)	54.1 (8.9)
25–34	463 (36.4)	69.4 (38.1)	102.2 (16.7)
35–44	376 (29.6)	36.4 (20.0)	89.7 (14.7)
45–54	249 (19.6)	25.2 (13.8)	115.3 (18.9)
55–64	101 (7.9)	22.3 (12.3)	103.5 (17.0)
65+	41 (3.2)	6.3 (3.5)	145.5 (23.8)
Education			
High school or less	38 (3.0)	18.2 (10.0)	62.1 (10.2)
Post-secondary	791 (62.2)	125.6 (69.0)	431.9 (70.8)
Graduate post-secondary	443(34.8)	38.3 (21.0)	116.3 (19.1)
Employment status			
Employed	1151 (90.5)	149.4 (82.0)	368.9 (60.5)
Unemployed	30 (2.4)	14.0 (7.7)	31.0 (5.1)
Student	43(3.4)	14.5 (8.0)	28.6 (4.7)
Retired	48 (3.8)	4.2 (2.3)	181.7 (29.8)
Born in Canada (yes)	805 (63.3)	117.4 (64.5)	434.7 (71.2)
Annual Household income			
< $35,000	61 (4.8)	35.4 (19.5)	72.7 (11.9)
$35,000 - $74,999	228 (17.9)	55.0 (30.2)	171.8 (28.1)
$75,000 - $149,999	465 (36.6)	51.7 (28.4)	187.3 (30.7)
$150,000+	346 (27.2)	18.2 (10.0)	68.5 (11.2)
No response	172 (13.5)	21.7 (11.9)	110.1 (18.0)
Has children living at home (yes)	289 (22.7)	27.2 (14.9)	79.2 (13.0)
Transportation Access and Behaviour			
Car share member (yes)	861 (67.7)	73.4 (40.3)	135.8 (22.3)
Access to personal car (yes)	817 (64.2)	129.2 (71.0)	482.6 (79.1)
Access to personal bicycle (yes)	888 (69.8)	96.5 (53.0)	347.1 (56.9)
Primary mode of transportation			
Drive	316 (24.8)	76.8 (42.2)	303.6 (49.7)
Transit	380 (29.9)	76.3 (41.9)	168.3 (27.6)
Walk	302 (23.7)	24.5 (13.5)	119.1 (19.5)
Bicycle	274 (21.5)	4.5 (2.5)	19.3 (3.2)
Bicycled in the past year, any type (yes)	–	96.3 (52.9)	220.3 (36.1)
Perceived safety of bicycling^a^			
Safe	1009 (79.3)	116.6 (64.1)	267.1 (43.8)
Neither safe nor unsafe		19.7 (10.8)	133.7 (21.9)
Dangerous		45.7 (25.1)	209.4 (34.3)
Perception of bicycle share in Vancouver^b^	–		
Good idea	–	170.9 (93.9)	414.8 (68.0)
Bad idea	–	7.3 (4.0)	145.1 (23.8)
Don’t know/Refused	–	3.8 (2.1)	50.4 (8.3)
Home and work location relative to Mobi by Shaw Go service area^c^			
Home and work outside	78 (8.0)	72.2 (41.8)	297.8 (50.3)
Home inside	240 (24.8)	42.2 (24.4)	137.4 (23.2)
Work inside	145 (15.0)	22.2 (12.8)	63.9 (10.8)
Home and work inside	506 (52.2)	36.2 (20.9)	93.0 (15.7)

^a^Based on the survey question, “Do you think that a public bike share program is a good or bad idea for Vancouver?”

^b^Based on the survey question, “Overall, how safe do you think cycling is in Vancouver?”

^c^Number of respondents with valid home and work locations: current users (*n* = 969), potential users (*n* = 173), unlikely users (*n* = 592). The Mobi by Shaw Go service area is defined as the area within 500 m of a bicycle share docking station.

### Potential users compared to unlikely users

Table 2 shows the results of the logistic regression models for demographic and transportation characteristics associated with being a potential user, compared to an unlikely user of the public bicycle share program. In the adjusted model, potential users were more likely to be employed (Odds ratio (OR): 2.04, 95% Confidence Interval (CI): 1.14, 3.67), and less likely to be aged 65+ compared to respondents aged 18–24 (OR: 0.18, 95% CI: 0.06, 0.53). Respondents with incomes less than $35,000 had four times the odds of being a potential user compared to respondents with incomes over $150,000. Transportation characteristics positively associated with being a potential user were having a car share membership (OR: 1.78, 95% CI: 1.17, 2.68), having bicycled in the past year (OR: 2.15, 95% CI: 1.30, 3.54), and using transit as a primary mode of transportation compared to walking (OR: 1.90, 95% CI: 1.05, 3.42). Importantly, potential users were less likely to own a personal bicycle than unlikely users (OR: 0.48, 95% CI: 0.29, 0.79), which suggests that there is interest for public bicycle share among those who may not bicycle regularly because they do not have easy or immediate access to a bicycle.

**Table 2:** Demographic and transportation characteristics associated with being a ‘potential user’ of the Vancouver public bicycle share program compared to a ‘unlikely user’, from a sub-sample of the 2017 Vancouver population survey (*n* = 792)

	Unadjusted OR (95% CI)	Adjusted OR^a^ (95% CI)
Sex (ref: Female)		
Male	1.05 (0.75, 1.47)	
Age (ref: 18–24 years)		
25–34	1.64 (0.87, 3.08)	1.31 (0.66, 2.61)
35–44	0.98 (0.51, 1.90)	0.96 (0.46, 1.98)
45–54	0.53 (0.26, 1.06)	0.61 (0.29, 1.30)
55–64	0.52 (0.25, 1.06)	0.68 (0.32, 1.45)
65+	0.10 (0.04, 0.28)	0.18 (0.06, 0.53)
Education (ref: High school or less)		
Post-secondary	0.99 (0.56, 1.75)	
Graduate post-secondary	1.12 (0.59, 2.14)	
Employment status (ref: Unemployed/Other^b^)		
Employed	2.99 (1.96, 4.56)	2.04 (1.14, 3.67)
Born in Canada (ref = No)		
Yes	0.73 (0.52, 1.04)	0.69 (0.47, 1.01)
Household Income (ref: >$150,000)		
$75,000 - $149,999	1.04 (0.57, 1.87)	1.12 (0.58, 2.14)
$35,000 - $74,999	1.20 (0.66, 2.17)	1.39 (0.72, 2.67)
< $35,000	1.83 (0.95, 3.50)	4.08 (1.92, 8.68)
No response	0.74 (0.37, 1.48)	1.16 (0.53, 2.55)
Has children living at home (ref: No)		
Yes	1.18 (0.74, 1.87)	
Carshare member (ref: No)		
Yes	2.36 (1.66, 3.36)	1.78 (1.17, 2.68)
Access to a personal car (ref: No)		
Yes	0.65 (0.44, 0.94)	
Access to a personal bicycle (ref: No)		
Yes	0.85 (0.61, 1.19)	0.48 (0.29, 0.79)
Primary mode of transportation (ref: Walk)		
Transit	2.21 (1.32, 3.69)	1.90 (1.05, 3.42)
Bicycle	1.13 (0.38, 3.30)	0.59 (0.19, 1.82)
Car	1.23 (0.75, 2.03)	1.69 (0.96, 2.99)
Bicycled in the past year, any type (ref: No)		
Yes	1.99 (1.42, 2.78)	2.15 (1.30, 3.54)
Perceived safety of cycling (ref: Unsafe)		
Neither safe nor unsafe	0.68 (0.38, 1.19)	0.62 (0.32, 1.18)
Safe	2.00 (1.36, 2.94)	1.71 (1.11, 2.64)
Home and work location relative to Mobi by Shaw Go service area (ref: Home and work outside)		
Home inside	1.27 (0.82, 1.95)	
Work inside	1.44 (0.83, 2.47)	
Home and work inside	1.61 (1.01, 2.56)	
Missing address	2.06 (0.89, 4.78)	

^a^Adjusted OR includes variables retained in multiple logistic regression

^b^Other includes students and retired respondents

### Motivators and deterrents

Motivators for potential users and deterrents for unlikely users are shown in Table 3. Among potential users, health was the most commonly selected motivator to using the public bicycle share program (selected by 47.0% of potential users). This was followed by motivators related to convenience, such as having docking stations near one’s home (45.5%) or destination (35.3%) and not owning a personal bicycle (41.0%). Motivators less commonly selected related to bicycle features and design.

**Table 3:** Motivators to bicycle share use among potential users (*n* = 182), ranked by the number of respondents that selected each item

Rank	Motivators	n (weighted)	% of total
1	For my health	86	47.0
2	Stations near home	83	45.5
3	I don’t have my own bicycle	75	41.0
4	Stations near destination	64	35.3
5	Cost is inexpensive	53	29.1
6	For fun	47	25.9
7	Helmets are provided	45	24.7
8	Convenience over other modes of transportation	35	19.5
9	Bicycles have a basket	34	18.6
10	System is easy to use	33	17.9
11	Bicycles have lights	33	17.9
12	Get to ride for free after paying membership fee	32	17.7
13	Bicycles have gears to help with hills	23	12.7
14	I like the appearance	9	4.8

Among those unlikely to use the program, the most common deterrents to using the program were preferring to ride a personal bicycle (46.9%) and the convenience of other transportation options (36.4%) (see Table 4). This was followed by barriers that pertain to bicycling in general, such as weather (35.8%), traffic (35.1%), and fear of injury from crashes or falls (23.2%). Cost was a deterrent to one-fifth of unlikely users. Other less common deterrents specific to the bicycle share program were not having stations near their destination, lack of knowledge about how to use public bicycle share, the weight of the bicycles, and not having enough bicycles at docking stations.

**Table 4:** Deterrents to bicycle share use among unlikely users (*n* = 610), ranked by the number of respondents that selected each item

Rank	Deterrents	n (weighted)	% of total
1	Prefer own bicycle	286	46.9
2	Less convenient than other types of transportation	222	36.4
3	Rain and bad weather	218	35.8
4	Traffic	214	35.1
5	Not interested in bicycling	192	31.4
6	Fear injury from crashes or falls	141	23.2
7	Cost is too expensive	122	20.1
8	No stations near home	118	19.3
9	Health concerns	106	17.3
10	Destinations are too far to bicycle	96	15.7
11	Time limits	92	15.1
12	Steep hills along my route	67	11.0
13	No stations near destination	58	9.5
14	I don’t know how to use the system	52	8.5
15	I don’t like having to wear a helmet	51	8.3
16	Bicycles are too heavy	35	5.8
17	No designated or separated bicycle lanes along my route	30	4.8
18	Not enough public bicycles at docking stations	15	2.4

## Discussion

This study examined the demographic and transportation characteristics of current, potential, and unlikely users of the public bicycle share program in Vancouver, Canada, as well as potential motivators and deterrents to public bicycle share use. Similar to trends observed in other cities [1, 4, 5], current public bicycle share users in Vancouver tended to be male, employed, and have higher educations and incomes as compared to non-users, and were more likely to use of active modes of transportation. Of the non-users, 23% were potential users and 77% were unlikely users. Potential users tended to be younger, have lower incomes, and were more likely to use public transit for their main mode of transportation, as compared to current and unlikely users. On a number of other sociodemographic and transportation characteristics, such as employment status, car share membership, car access, and perceived safety of cycling, the profile of potential users was somewhere in between current and unlikely users.

As of fall 2017, estimates suggest the proportion of the population that had used the public bicycle share program is 6.2% [23]. Among those who have not used the program, the majority (74%) think that a public bicycle share is a good idea for Vancouver, and nearly one in four indicated they are likely to use public bicycle share within the next year. This suggests that there is considerable opportunity to increase population level uptake of public bicycle share. The challenge for public bicycle share operators is translating intention into action. Intention is an important part of behavior change models [24, 25], however similar to other intentions such as eating healthier foods or exercising more, intention does not necessarily translate into action without the proper conditions or incentive to change, referred to as the ‘intention-behaviour gap’ [26–28]. Social marketing is one approach that can help lessen the gap between intention and action. This involves understanding the potential consumer’s viewpoint and designing a product or service to suits their needs [10].

The marketing mix, also known as the 4 Ps, are considered the core elements of a social marketing strategy, and include *product, price, place, and promotion* [29]*.* The *product* refers to the object or service being offered, and the benefits associated with the product [29]. In the case of public bicycle share, the product is a service which allows users to rent and return bicycles at docking stations throughout designated areas of a city (Table 5). Amongst the potential user group, health benefits and having stations located near their home and work were the most commonly cited motivators that would influence them to use public bicycle share. *Price* refers to the perceived costs of the product or service being offered and includes both monetary costs and non-monetary costs, such as time and effort [29]. Our findings showed that potential users were much more likely to have lower incomes compared to current users, and that cost was cited as a barrier among 20% of unlikely users. In spring 2018, Mobi by Shaw Go announced a one-year pilot to offer discounted memberships ($20) to low income residents [30]. Continuing this pilot, reducing the cost of regular memberships, and offering a cheaper single trip rate and free trial days could reduce the barrier for non-users to try public bicycle share. In addition, given that potential users were more likely to use transit and belong to car share programs compared to unlikely users, integrating public bicycle share payment with transit passes and car share memberships could be an effective strategy, and has been done in other cities such as in Montréal and Pittsburg [31, 32]. *Place* refers to where and when the consumer can access the product or service [29]. Previous studies have shown that those who live and work in close proximity to public bicycle share docking stations are more likely to use the program [13, 14, 33, 34]. Public bicycle share service areas in many cities tend to disproportionately serve higher socioeconomic status neighbourhoods [35, 36], and Vancouver is no exception [37]. This may explain, in part, why current public bicycle share users in Vancouver were more likely to be of higher socioeconomic status compared to non-users. In addition to station distribution, public bicycle share uptake is dependent on a city’s efforts in providing cycling infrastructure in the areas where the public bicycle share service area is located [38]. Other than cost, station distribution, and preference for one’s own bicycle, the common deterrents to public bicycle share use related to barriers to bicycling more generally, such as rain and bad weather, traffic, lack of interest in bicycling, and fear of injury from crashes or falls. This emphasizes that a city’s efforts to provide safe bicycle infrastructure and promote bicycling as a transportation option are important for public bicycle share uptake. Finally, *promotion* refers to the communication and advertising strategies used to promote the product or service [39]. The profile of the potential user group identified in this study and their motivators can help inform the development of advertising strategies.

**Table 5:** The four “Ps” of social marketing applied to a public bicycle share program

Product	Public bicycle share service, which allows users to rent and return bicycles at designated docking stations throughout a city
Price	Reduce cost of memberships Offer a single trip pass option Free trial days Integrate payment with transit and car share programs
Place	Expand service area Provide safe bicycle infrastructure in areas where public bicycle share stations are located
Promotion	Pop-up booths at transit stations Advertising on public transit

### Strengths and limitations

This exploratory study used data from a public bicycle share member survey and a population-based survey to better understand the profiles of current, potential, and unlikely users of a public bicycle share program in Vancouver, Canada. The use of a population-based survey allowed us to identify demand for public bicycle share among non-users at the population level, providing valuable information about who is in the ‘near market’ for the public bicycle share program and who is unlikely to use it. Our findings can help inform public bicycle share operators about the importance of station distribution, cost, and marketing and outreach efforts for the success of their program.

There are several limitations worth noting. The demographic characteristics of current users reflects those of the members who completed the Mobi member survey (response rate 29.4%) rather than all members of the public bicycle share program. Demographic information is not collected for all members so we cannot assess the generalizability of our sample in terms of demographic characteristics, however, survey respondents did have a slightly higher frequency of bicycle share use (average of 10.6 trips per month) as compared to the average Mobi member (7.7 trips per month). We did not consider frequency of public bicycle share use for current or potential users, however, future studies could stratify users based on their frequency of use, or intended frequency of use for potential users.

The survey sample in the Vancouver panel survey was representative of the Vancouver population based on age and sex, but underrepresented immigrants, and residents with lower incomes and educations. This is a common challenge in surveys. To categorize potential and unlikely users we used a question that asked respondents to indicate their likelihood of use within the next year given that station locations were accessible to them. This could relate to access at work, home, or other common places visited, however, there may have been differences in interpretation. We also had small sample size of potential users (*n* = 182), which resulted in wide confidence intervals in the logistic regression model in some cases.

We asked respondents to select all the reasons that would influence their decision to use or not use public bicycle share, but did not ask them to weight their relative influence. Thus, the most commonly selected factors presented here should not necessarily be conflated with the most important factors to influence behavior change. For example, health was the most commonly selected motivator among potential users. Although health may be a desired benefit, health on its own is a poor motivator for influencing behavior change [40]. Also, respondents may not have had sufficient knowledge about the program to assess all motivators and deterrents, such as program cost or time limits.

Finally, the findings from this study reflect the likelihood of public bicycle share use in the Vancouver population. It is difficult to assess the generalizability of these findings to other cities. However, the demographic profile of current users in Vancouver is similar to the demographic profile identified in other cities, which could suggest that there are also similarities to the profiles of potential and unlikely users identified in this study.

## Conclusion

Public bicycle share programs are widely touted as having the potential to reduce the public health burden associated with physical inactivity and also reduce air pollution, greenhouse gas emissions, and motor vehicle traffic. However, public bicycle share programs in many cities, including Vancouver, tend to appeal to a higher socioeconomic status segment of the population that primarily use active modes of transportation for day to day travel. In order to meaningfully contribute to shifts towards active and sustainable modes of transportation, and to do so equitably, public bicycle share programs need to appeal to a broader population. Our results suggest there is interest for the public bicycle share program among non-users, particularly among those who are younger, have lower household incomes, and use public transit. To reach currently underrepresented lower income populations, reducing the cost and expanding the service area to lower income neighbourhoods are likely to help. Findings from this study can help inform targeted marketing and outreach to increase public bicycle share uptake in the population.

## Additional files

Additional file 1: Question inventory for the 2017 Mobi Member Shaw Go User Survey. (PDF 207 kb)

Additional file 2: Question inventory for the 2017 Vancouver Public Bike Share Population Survey. (PDF 224 kb)

## Abbreviations

AIC: Akaike information criterion
CI: Confidence Interval
OR: Odds ratio
