# Supplementary material for: Who is in the near market for bicycle sharing? Identifying current, potential, and unlikely users of a public bicycle share program in Vancouver, Canada
Source: BMC Public Health. 2018 Nov 29;18:1326. doi: 10.1186/s12889-018-6246-3 (PMC6267823; doi:10.1186/s12889-018-6246-3)
Supplement: Supplementary file 1 — Question inventory for the 2017 Mobi Member Shaw Go User Survey. (PDF 207 kb) [file 12889_2018_6246_MOESM1_ESM.pdf]

Additional file 1: Mobi by Shaw Go User Survey
Fall 2017 Survey - Question Inventory

QUESTIONS	RESPONSE CATEGORIES (Refuse and Don't know/not sure for every question)
SECTION A: TRAVEL BEHAVIOUR AND PHYSICAL ACTIVITY	
Q1. Overall, which mode of transportation do you use most often to get around?	private motor vehicle, car share, bus or seabus, SkyTrain, bicycle, walk, motorcycle, taxi, other
Q2. Overall, how safe do you think cycling is in Vancouver? Would you say it is:	very safe, somewhat safe, neither safe nor unsafe, somewhat dangerous, very dangerous
Q3. In the past 3 months, how often did you typically travel using a Mobi by Shaw Go bicycle?	4 or more days per week, 1-3 days per week, 1-3 days per month, less than once per month
Q4. In the past 3 months, how often did you typically travel using your own bicycle?	4 or more days per week, 1-3 days per week, 1-3 days per month, less than once per month
Q5. In the past 7 days, did you do any of the following?<br>a. WALKING for recreation, health, or fitness? (including walking your dog)<br>b. WALKING to travel to or from work, school, or another destination (one-way travel)<br>c. CYCLING for recreation, health, or fitness? (Mobi by Shaw Go or personal bicycle)<br>d. CYCLING to travel to or from work, school, or another destination (one-way travel on a Mobi by Shaw Go or personal bicycle)	For each sub-question:<br>[# of time this past week]<br>[# of minutes this past week]
SECTION B: MOBI BY SHAW GO USE	
Q6. How did you first learn about Mobi by Shaw Go?	employer/co-worker/information at work, friend/family member, community event, social media, newspaper/magazine/radio/TV, saw a Mobi by Shaw Go station on the street, local government, brochure, Mobi by Shaw Go website, other
Q7. When did you first become a Mobi by Shaw Go member?	[month, year]
Q8. As a result of your use of Mobi by Shaw Go, do you use each of the following types of travel options more often, less often, or about the same as before you joined Mobi by Shaw Go?<br>a. Private motor vehicle<br>b. Car share<br>c. Bus or seabus<br>d. SkyTrain<br>e. Personal bicycle<br>f. Walk<br>g. Motorcycle<br>h. Taxi	much more often, somewhat more often, about the same, somewhat less often, much less often, not applicable/never used

Q9. Which of the following best describes your TYPICAL helmet use when riding a Mobi by Shaw Go bicycle?	I wear the provided Mobi by Shaw Go bicycle helmet (WITH the helmet liner), I wear the provided Mobi by Shaw Go helmet (WITHOUT the helmet liner), I wear my personal bicycle helmet, I don't typically wear a helmet
Q10. What is your main reason for not typically wearing a helmet when riding a Mobi by Shaw Go bicycle?	discomfort, poor fit, too hot, sanitary concerns with the shared helmets, helmet was wet, helmet was damaged, I find helmets unattractive, I prefer wearing a hat suited to the weather conditions (e.g., sunhat, winter hat), I don't need a helmet because I ride slowly or in quiet areas, I don't usually wear a helmet, other
Q11. Since joining Mobi by Shaw Go, how many trips have you made on a Mobi by Shaw Go bicycle? By trips we mean one-way trips (e.g., from home to work).	0, 1, 2, 3, more than 3
The following questions ask about the last 3 trips you made using Mobi by Shaw Go.	[sub-questions repeated up to 3 times for each trip]
i What was the trip purpose?	transportation to or from work, transportation as part of work activities, transportation to or from school, leisure or fun, exercise, shopping or to run other errands, socializing – meeting with friends or family, other
ii Would you have made this trip if Mobi by Shaw Go was not available?	yes, no
iii If yes, what mode would you have used?	private motor vehicle, car share, bus or seabus, SkyTrain, personal bicycle, walk, motorcycle, taxi, other
iv What other modes of transportation did you combine with Mobi by Shaw Go on this trip? (select all that apply)	none, private motor vehicle, car share, bus or seabus, SkyTrain, personal bicycle, walk, motorcycle, taxi, other
SECTION C: MOTIVATORS AND BARRIERS	
Q13. In general, which of the following reasons motivated your decision to use Mobi by Shaw go?<br>a. I have fun riding Mobi by Shaw Go bicycles<br>b. I don't have my own bicycle, but Mobi by Shaw Go means I can now cycle<br>c. I get around more easily or faster than other types of transportation<br>d. For my health<br>e. My concern about the environment<br>f. Mobi by Shaw Go is a good way to spend less money on transportation<br>g. I don't have to worry about the bicycle being stolen	[select all that apply]

h. I can ride one-way or for just part of my trip<br>i. I like that my membership includes unlimited rides<br>j. There are stations near where I live<br>k. There are stations near where I am going<br>l. I find the system easy to use<br>m. I find parking a car is difficult/expensive<br>n. I have a discounted or free membership<br>o. It is an employer benefit<br>p. I want to support the Vancouver public bicycle share system<br>q. Other	
Q14. In general, which of the following reasons prevent you from using Mobi by Shaw Go more often?<br>a. I prefer to ride my own bicycle most of the time<br>b. Other types of transportation are more convenient<br>c. The cost to use Mobi by Shaw Go is too expensive<br>d. The Mobi by Shaw Go time limit for free riding is too short for me<br>e. I don't like having to wear a helmet<br>f. There are no stations near where I live<br>g. There are no stations near where I am going (work/school or other destinations)<br>h. Stations are often moved<br>i. Stations are not working<br>j. There are not enough bicycles/free docks at docking stations<br>k. There are steep hills along my route<br>l. My destinations are too far to bicycle<br>m. It takes too long to sign the bicycles in and out<br>n. Mobi by Shaw Go bicycles are too heavy<br>o. Mobi by Shaw Go bicycles often have maintenance problems<br>p. Mobi by Shaw Go gearing doesn't work on Vancouver terrain<br>q. I don't like to ride in traffic<br>r. I fear injury from crashes or falls<br>s. There are not separated bicycle lanes along my route<br>t. I don't like to ride in rain and bad weather<br>u. Mobi by Shaw Go bicycles are uncomfortable/the wrong size<br>v. Helmet fit or sanitation issues<br>w. Not enough cargo space to transport goods, children, or pets<br>x. None of the above – I am happy with how much I use it<br>y. Other	[select all that apply]
Q15. Where specifically would you like Mobi by Shaw Go to install another station (e.g., street, neighbourhood, landmark)	open ended response
SECTION C: MOBI BY SHAW GO CUSTOMER SERVICE	

Q16a. How satisfied are you with your Mobi by Shaw Go membership?	very satisfied, satisfied, neither satisfied nor dissatisfied, dissatisfied, very dissatisfied, no opinion
Q16b. Please rate how easy or difficult it was for you to understand the following when using Mobi by Shaw Go?<br>a. Finding a bicycle or station<br>b. Plan options and pricing information<br>c. Overage fees<br>d. Locking a bicycle	very easy, somewhat easy, neutral, somewhat difficult, very difficult, not applicable
Based on your experience using Mobi by Shaw Go bicycles, please rate the following items:<br>Q17. Features of bicycles and stations<br>a. Condition and cleanliness of bicycles<br>b. The way to report problems with bicycles<br>c. Map at stations<br>d. Lighting at stations<br>Q18a. Website<br>a. Website general functionality<br>b. Registration process through website<br>c. Station map on website<br>Q18b. App<br>a. App general functionality<br>b. Registration process through app<br>c. Station map on app<br>Q19. The Call Centre<br>a. Call centre wait times<br>b. Customer service representative's ability to solve issues	Rate from 1-5 (Excellent-Poor)
SECTION D: CYCLING INCIDENTS	
Q20. How many times in the past 3 months have you been involved in a crash or fall (for any reason) while riding your own bicycle in the city	[# of times]
Q21. How many times in the past 3 months have you been involved in a crash or fall (for any reason) while riding a Mobi by Shaw Go bicycle in the city?	[# of times]
We would like to know more information about your crashes or falls in the city on your own bicycle or on a Mobi by Shaw Go bicycle in the past 3 months. *If you have had more than 3 crashes or falls in the past 3 months, please provide information on the most 3 incidents.*	Q22 repeated up to 3 times for each reported crash
Q22a. What was the month of the crash or fall	June 2017, July 2017, August 2017, September 2017
Q22b. Did your crash or fall involve any of the following?<br>a. Collision with a vehicle door being opened (dooring)<br>b. Other collision with a motor vehicle (including car, SUV, truck, bus, motorcycle)<br>c. Collision with another cyclist<br>d. Collision with a pedestrian<br>e. Hitting a hazard on the route (such as a train track, post, pothole, curb, slippery surface)	[select all that apply]

f. A fall when trying to avoid a collision<br>g. You being distracted<br>h. A mechanical issue (brakes, gears, pedals)<br>i. None of the above	
Q22c. Were you on a Mobi bicycle at the time of the crash or fall?	yes, no
Q22d. Did any of the following result from your crash or fall?<br>a. I was injured<br>b. I visited a hospital emergency department because I was injured<br>c. I was admitted to hospital (overnight stay in a department other than emergency)<br>d. None of the above	[select all that apply]
Q22e. Was the crash reported to any of the following?<br>a. Insurance Corporation of British Columbia (ICBC)<br>b. The police<br>c. Mobi by Shaw Go (if on a Mobi bicycle)<br>d. BikeMaps.org<br>e. I did not report my crash<br>f. Other	[select all that apply]
SECTION E: DEMOGRAPHICS	
Q23. Do you have a driver's licence?	yes, no
Q24. What car share services are you part of?	Car2Go, Modo, Zipcar, Evo, other
Q25. How many of the following vehicles are kept in your household?<br>a. Bicycles for adults (including tandems, trikes, or cargo bicycles without electric assist)<br>b. E-bicycles (including all bicycles with any form of assistance)<br>c. Bicycles for children<br>d. Cars, vans, or trucks<br>e. Motorcycles	[# 0-20]
Q26. How many people, including yourself, live in your household?<br>a. Children aged under 5<br>b. Children between 5 and 15<br>c. Adults aged 16 and over (including yourself)	[# 0-10]
Q27. Are you…?	female, male, other
Q28. Age	16-18, 18-24, 25-34, 35-44, 45-54, 55-64, 65+
Q29. What is the highest level of education you have completed?	some high school or less, graduated high school, college/vocational/technical, some university, graduated university, graduate degree (e.g., masters), other
Q30. What is your postal code of your home? OR If you do not know the postal code, please provide the nearby cross streets.	[postal code or cross streets]
Q31. What is your current employment status?	work for pay full-time (≥30 hours/week), working for pay part-time (<30 hours/week),

Additional file 1: Mobi by Shaw Go User Survey
Fall 2017 Survey - Question Inventory

	seasonal/temporary work, homemaker, student, retired, unemployed
Q32. What is your postal code of your main place of work or study? OR if you do not know the postal code, please provide the name of your workplace or school and nearby cross streets.	[postal code or address or cross streets]
Q33. Were you born in Canada?	yes, no
Q31. Vancouver residents come from many different backgrounds. How would you describe yourself? (Select up to two options)	North American, African, Latin American, Asian, European, Middle Eastern, Oceanic, Indigenous Peoples in Canada, other
Q32. In general, for someone your age, would you say your health is:	excellent, very good, good, fair, poor
Q33. Which of the following best describes your total annual household income before taxes?	under $20,000, $20,000 up to $34,999, $35,000 up to $49,999, $50,000 up to $74,999, $75,000 up to $99,999, $100,000 up to $149,999, $150,000 up to $199,999, Over $200,000
