# Supplementary material for: Who is in the near market for bicycle sharing? Identifying current, potential, and unlikely users of a public bicycle share program in Vancouver, Canada
Source: BMC Public Health. 2018 Nov 29;18:1326. doi: 10.1186/s12889-018-6246-3 (PMC6267823; doi:10.1186/s12889-018-6246-3)
Supplement: Supplementary file 2 — Question inventory for the 2017 Vancouver Public Bike Share Population Survey. (PDF 224 kb) [file 12889_2018_6246_MOESM2_ESM.pdf]

Additional file 2: Vancouver Population Public Bike Share Online Survey
Fall 2017 Survey - Question Inventory

**QUESTIONS**	**RESPONSE CATEGORIES** (Refuse and Don't know/not sure for every question)
SCREENING QUESTIONS	
S0. Where in British Columbia do you live?	City of Vancouver, outside the City of Vancouver but within the Greater Vancouver Regional District, Vancouver Island, elsewhere in British Columbia
S1. What neighbourhood do you live in?	Arbutus-Ridge, Downtown, Dunbar-Southlands, Fairview, Grandview-Woodland, Hastings-Sunrise, Kensington-Cedar Cottage, Kerrisdale, Killarney, Kitsilano, Marpole, Mount Pleasant, Oakridge, Renfrew-Collingwood, Riley Park, Shaughnessy, South Cambie, Strathcona, Sunset, Victoria-Fraserview, West End, West Point Grey
S2. What year were you born?	[Year – code age categories accordingly]
SECTION A: TRAVEL BEHAVIOUR AND PHYSICAL ACTIVITY	
Q1. Overall, which mode of transportation do you use most often to get around?	car/truck, transit bus, SkyTrain, seabus, bicycle, walk, motorcycle, taxi, other
Q2. Think about your journeys to and from work (e.g., travel to and from your place of work, accompanying your spouse to and from their work).<br>a. How often did you make such a journey over the last 7 days?	[# of times]
b. How much time in total over the last 7 days did you spend travelling to and from work by:<br>i Walking<br>ii Bicycle<br>iii Bus<br>iv SkyTrain<br>v Car<br>vi Car share<br>vii Other	[# of minutes]
Q3. Think about your business journeys, by which we mean any journeys in the course of your work or on employer's business (e.g. travel to and from meetings, making deliveries, etc.)<br>a. How often did you make such a journey over the last 7 days?	[# of times]
b. How much time in total over the last 7 days did you spend travelling to and from work by:<br>i Walking	[# of minutes]

ii Bicycle<br>iii Bus<br>iv SkyTrain<br>v Car<br>vi Car share<br>vii Other	
Q4. Think about your journeys to and from a place of study (e.g. travel to and from your university or college) or to and from school (e.g. if you accompany a child to and from school).<br>a. How often did you make such a journey over the last 7 days?	[# of times]
b. How much time in total over the last 7 days did you spend travelling to and from work by:<br>i Walking<br>ii Bicycle<br>iii Bus<br>iv SkyTrain<br>v Car<br>vi Car share<br>vii Other	[# of minutes]
Q5. Think about your journeys for shopping and personal business (e.g. food shopping, non-food shopping, window-shopping, visiting a doctor, bank, solicitor or estate agents, visiting a relative in hospital, or accompanying someone else to a doctor, hospital etc.).<br>a. How often did you make such a journey over the last 7 days?	[# of times]
b. How much time in total over the last 7 days did you spend travelling to and from work by:<br>i Walking<br>ii Bicycle<br>iii Bus<br>iv SkyTrain<br>v Car<br>vi Car share<br>vii Other	[# of minutes]
Q6. Think about your journeys to visit friends and relatives and for other social activities. (e.g. a journey to and from the cinema or other entertainment facilities<br>a. How often did you make such a journey over the last 7 days?	[# of times]
b. How much time in total over the last 7 days did you spend travelling to and from work by:<br>i Walking<br>ii Bicycle<br>iii Bus<br>iv SkyTrain<br>v Car<br>vi Car share<br>vii Other	[# of minutes]
Q7. In the last 7 days, did you do any walking for recreation, health or fitness	yes, no
a. In the last 7 days, how many times did you walk for recreation, health, or fitness?	[# of times]

b. Please estimate the total time you spent walking for recreation, health, or fitness (e.g., 2 times x 20 minutes = 40 minutes)	[# of minutes]
Q8. In the last 7 days did you do any cycling for recreation, health or fitness?	yes, no
a. In the last 7 days, how many times did you cycle for recreation, health or fitness	[# of times]
b. Please estimate the total time you spend cycling for recreation, health or fitness in the last 7 days (e.g., 2 times x 20 minutes = 40 minutes)	[# of minutes]
Q9. In the last 7 days, did you do any moderate to vigorous intensity physical activity in your leisure time? *This could by any activity that made you sweat, such as jogging, playing sports, or the gym.*	yes, no
a. In the last 7 days, how many times did you do moderate to vigorous intensity physical activities in your leisure time?	[# of times]
b. Please estimate the total time you spent do moderate to vigorous intensity physical activities in your leisure time in the last 7 days (e.g.2 times x 20 minutes = 40 minutes).	[# of minutes]
Q10. In the last 7 days, did you do any moderate to vigorous intensity physical activities as part of your job? This could be any activity that made you sweat, such as jogging, playing sports, or the gym.	yes, no
a. In the last 7 days, how many times did you do moderate to vigorous intensity physical activities as part of your job?	[# of times]
b. Please estimate the total time you spent do moderate to vigorous intensity physical activities as part of your job in the last 7 days (e.g.2 times x 20 minutes = 40 minutes).	[# of minutes]
SECTION B: CYCLING BEHAVIOUR	
Q11. Overall, how safe do you think cycling is in Vancouver? Would you say it is:	very safe, somewhat safe, neither safe nor unsafe, somewhat dangerous, very dangerous
Q12. In the previous 12 months, have you used a bicycle?	yes, no
Q12a. [ASK IF Q12 = yes] How often do you typically travel by bicycle?	4 or more days per week, 1-3 days per week, 1-3 days per month, less than once per month
Q12b. [ASK IF Q12 = yes] On your last trip by bicycle, did you wear a helmet?	yes, no
Q13a. Would you consider using a bicycle in future?	yes, no
Q14. On a 4-point scale, with 1 being strongly disagree and 4 being strongly agree, how much would you agree with the following statement: "I would like to travel by bicycle more than I do now."	strongly agree, somewhat agree, somewhat disagree, strongly disagree
Q15a. Have you walked or bicycled on the new Arbutus Greenway in the past 12 months?	yes, no
Q15b. Have you walked or bicycled on the Point Grey Road Seaside Greenway in the past 12 months?	yes, no
SECTION C: PUBLIC BIKE SHARE	
Q16. Have you ever heard of a public bike share program	yes, no

Additional file 2: Vancouver Population Public Bike Share Online Survey
Fall 2017 Survey - Question Inventory

Q17. Do you think a public bike share program is a good or bad idea for Vancouver?	very good idea, somewhat good idea, somewhat bad idea, very bad idea
Q18. Have you seen a public bike share station in Vancouver?	yes, no
Q19. Have you seen anyone riding a public bike share bicycle in Vancouver?	yes, no
Q20. Have you ever ridden a public bike share bike in Vancouver?	yes, no
Q21. How likely would you be to use public bike share in Vancouver at some point in the next year, given that station locations are accessible to you?	very likely, somewhat likely, not very likely, not at all likely
Q22a. [ASK IF Q16 = very likely or somewhat likely] Which of the following reasons influence your decision to use the Vancouver public bike share program?<br>a. I have fun riding the bike share bicycles<br>b. I don’t have my own bike, but public bike share means I can now cycle<br>c. Other types of transportation are less convenient<br>d. For my health<br>e. The cost to use the public bike share is inexpensive<br>f. I like being able to ride for free after paying my membership fee<br>g. I like that the public bike share program provides a helmet<br>h. There are stations near where I live<br>i. There are stations near where I am going<br>j. I find the system easy to use<br>k. The bike share bikes have lights and seem very safe<br>l. There are gears that help me with hills<br>m. They have a basket up front to hold my things<br>n. I like the way they look<br>o. Other	[select all that apply]
Q22b. [ASK IF Q16 = not very likely or not at all likely] Which of the following reasons influence your decision to not use the Vancouver public bike share program?<br>a. I am not interested in cycling<br>b. I prefer to ride my own bicycle<br>c. Other types of transportation are more convenient<br>d. Health and age issues prevent me from riding a bike<br>e. The cost to use the public bike share program is too expensive<br>f. The public bike share program’s time limitations are not ideal for me<br>g. I don’t like having to wear a helmet<br>h. There are no stations near where I live<br>i. There are not stations near where I am going<br>j. I fear injury from crashes or falls<br>k. I don’t understand how to use the system<br>l. The public bike share bikes are too heavy<br>m. There are steep hills along my route<br>n. There are not enough public bicycles at docking stations<br>o. My destinations are too far to bike<br>p. I don’t like to ride in traffic	[select all that apply]

q. There are not designated or separated bicycle lanes along my route<br>r. I don't like to ride in rain and bad weather<br>s. Other	
SECTION D: PUBLIC BIKE SHARE USERS (Asked only to those who have used the public bike share program in Vancouver [Q36 = yes])	
Q23. On average, how many times per day, week, month, or year do you use a public bike share bicycle?	[# of times]
Q24. On average per trip, how many minutes or hours do you use a public bike share bicycle?	[# of minutes or hours]
Q25. When you use public bike share bicycles, what type of trip does it TYPICALLY involve?<br>a. Transportation to or from work or school<br>b. Leisure or fun<br>c. Exercise<br>d. Shopping or to run other errands<br>e. Socializing – meeting with family or friends<br>f. Trips for work<br>g. Other	[select all that apply]
Q26. When you use public bike share bicycles, which of the following modes do you TYPICALLY integrate into your travel?<br>a. Walking<br>b. Transit-bus<br>c. Transit-SkyTrain<br>d. Transit-seabus<br>e. Private motor vehicle<br>f. Car share<br>g. Taxi<br>h. Other	[select all that apply]
Q27. What type of transportation would you have used to make these trips if public bike share was not available?<br>a. Walking<br>b. Transit-bus<br>c. Transit-SkyTrain<br>d. Private motor vehicle<br>e. Car share<br>f. Taxi<br>g. Personal bicycle<br>h. Other<br>i. These were trips I would not normally have made otherwise	[select all that apply]
Q28. Which of the following best describes your TYPICAL helmet use when riding a public bike share bicycle?	I wear the provided Mobi by Shaw Go helmet, I wear my personal helmet, I don't typically wear a helmet
Q29. What is your main reason for not always using a helmet when riding a public bike share bicycle?	discomfort, poor fit, too hot, sanitary concerns with the shared helmet, helmet was wet or damaged, I find helmets unfashionable, I don't need a helmet because I ride slowly or in

	quiet areas, I don't usually wear a helmet
SECTION E: SOCIAL PARTICIPATION	
Q30. How would you describe your sense of belonging to your local community? Would you say it is:	Very strong, somewhat strong, somewhat weak, very weak
Q31. How often do you…<br>a. Say hello to a neighbour?<br>b. Stop and have a chat with a neighbour?<br>c. Visit a neighbour, or receive a visit from a neighbour?<br>d. Go somewhere (e.g., to a shop; restaurant), together with a neighbour?<br>e. Ask help/advice from or do you help/give advice to a neighbour yourself?	[# of times per week/month/year]
Q32. If you lost a wallet or purse that contained two hundred dollars, how likely is it to be returned with the money in it, if it was found by:<br>a. Someone who lives close by?<br>b. A complete stranger	very likely, somewhat likely, not at all likely
SECTION F: CYCLING INCIDENTS	
Q33. How many times in the past 3 months have you been involved in a crash or fall (for any reason) while riding your bike in the city?	[# of times]
Q34. We would like more information about your crashes or falls in the city in the past 3 months. If you have had more than 3 crashes or falls in the past 3 months, please provide information on the 5 most recent incidents	
a. Month of crash or fall	[month]
b. Was it reported to ICBC?<br>c. Was it reported to the police?<br>d. Were you injured?<br>e. Did you visit a hospital emergency department because you were injured?<br>f. Were you admitted to a hospital? (overnight stay in a department other than emergency)<br>*Did your crash or fall involve any of the following?*<br>g. Collision with a vehicle door being opened<br>h. Other collision with a motor vehicle (including car, SUV, truck, bus, motorcycle)<br>i. Collision with another cyclist<br>j. Collision with a pedestrian<br>k. Hitting a hazard on the route (such as a train track, post, pothole, curb, slippery surface)<br>l. A fall when trying to avoid a collision<br>m. You being distracted<br>n. A mechanical issue (brakes, gears, pedals)<br>o. Did this crash occur while you were riding a Vancouver public bike share bicycle?	yes, no
SECTION G: DEMOGRAPHICS	
Q35. Do you have a driver's license?	yes, no

Additional file 2: Vancouver Population Public Bike Share Online Survey
Fall 2017 Survey - Question Inventory

Q36. How many of the following vehicles are kept in your household?<br>a. Bicycles for adults<br>b. Bicycles for children<br>c. Cars or vans<br>d. Motorcycles	[# 0-20]
Q37. What car share services are you part of?	Car2Go, Modo, Zipcar, Evo, other
Q38. How many people, including yourself, live in your household?<br>a. Children aged under 5<br>b. Children between 5 and 15<br>c. Adults aged 16 and over	[# 0-10]
Q39. How long have you lived in your current home residence?	[# of years]
Q40. What is your postal code of your home? OR If you do not know the postal code, please provide nearby cross streets to your home.	[postal code OR cross streets]
Q41. Are you…?	female, male
Q42. What is the highest level of education you have completed?	some high school or less, graduated high school, college/vocational/technical, some university, graduated university, graduate degree (e.g., masters), other
Q43. What is your current employment status?	work for pay full-time (≥30 hours/week), working for pay part-time (<30 hours/week), seasonal work, homemaker, student, retired, unemployed
Q44. What is your postal code of your main place of work or study? OR if you do not know the postal code, please give the address, or nearby cross streets of your place of work or study.	[postal code or address or cross streets]
Q45. Were you born in Canada?	yes, no
Q46. Vancouver residents come from many different backgrounds. How would you describe yourself? (Select up to two options)	North American, African, Latin American, Asian, European, Middle Eastern, Oceanic, Other
Q47. In general, for someone your age, would you say your health is:	excellent, very good, good, fair, poor
Q48. Which of the following best describes your total annual household income before taxes?	under $20,000, $20,000 up to $34,999, $35,000 up to $49,999, $50,000 up to $74,999, $75,000 up to $99,999, $100,000 up to $149,999, $150,000 up to $199,999, Over $200,000
